# Clinical Consequences for Individuals Treated with Tocilizumab for Serious COVID-19 Infection

**DOI:** 10.3390/healthcare11040607

**Published:** 2023-02-17

**Authors:** Al Shaimaa Ibrahim Rabie, Hager Salah, Amira S. A. Said, Ahmed Hassan Shaaban, Lamya Mohamed Abdou, Doaa Mahmoud Khalil, Zelal Kharaba, Hala Afifi, Mahmoud R. Sofy, Eman M. I. Youssef, Eman S. M. Bayoumy, Raghda R. S. Hussein

**Affiliations:** 1 Clinical Pharmacy Department, Fayium Oncology Center, Fayium 63511, Egypt; 2 Clinical Nutrition Department, Fayium Health Insurance Authority, Fayium 63511, Egypt; 3 Pharmaceutical Services Department, King Hamad University Hospital, Al Sayh 24343, Bahrain; 4 Department of Clinical Pharmacy, Faculty of Pharmacy, Beni-Suef University, Beni-Suef 62514, Egypt; 5 Department of Clinical Pharmacy, College of Pharmacy, Al Ain University, Al Ain, Abu Dhabi 64141, United Arab Emirates; 6 Clinical Oncology Department, Faculty of Medicine, Beni-Suef University, Beni-Suef 62514, Egypt; 7 Internal Medicine Department, Faculty of Medicine, Beni-Suef University, Beni-Suef 62514, Egypt; 8 Public Health and Community Medicine Department, Faculty of Medicine, Beni-Seuf University, Beni-Suef 62514, Egypt; 9 AAU Health and Biomedical Centre, Al Ain University, Al Ain, Abu Dhabi 64141, United Arab Emirates; 10 Faculty of Medical Sciences, Newcastle University, Newcastle Upon Tyne NE2 4HH, UK; 11 College of Dental Surgery, City University Ajman, Ajman 18484, United Arab Emirates; 12 Botany and Microbiology Department, Faculty of Science, Al-Azhar University, Cairo 11884, Egypt; 13 Department of Biochemistry, Faculty of Medicine, Al-Azhar University, Cairo 11884, Egypt; 14 Department of Biochemistry, College of Medicine, Taif University, Taif 11099, Saudi Arabia; 15 Clinical Pharmacy Department, Faculty of Pharmacy, October 6 University, 6 October City 12858, Egypt

**Keywords:** COVID-19, cytokine storm, tocilizumab

## Abstract

There seem to currently be no therapeutic medications found for the severe coronavirus infection in 2019 (COVID-19). In light of this, it has been hypothesized that the immunomodulatory treatment known as tocilizumab can lessen the inflammatory response that occurs in the respiratory system, speed up the process of clinical benefit, lower the risk of death, and avert the need for ventilators. This randomized controlled trial (RCT) studied patients with a proven infection of SARS-CoV-2 and hyperinflammatory reactions. The inclusion criteria included fever (body temperature > 38 °C), pulmonary infiltrates, or supplemental oxygen. The patients received either conventional treatment with one dose of either tocilizumab (8 mg per kilogram of body weight) or conventional treatment only. The subjects were randomized to receive either treatment with a 1:1 ratio. A time-to-event test was conducted to determine the time to intubation or death. There was an insignificant difference between the investigated groups regarding the time to death, time to mechanical ventilation, and percentage of deaths. The conventional group’s median (IQR) hospital length of stay was 4 (3–6) days, whereas the tocilizumab therapy group was 7 (4.75–10) days. There was a substantial difference in the mechanical ventilation rates in both groups, which were 17 (34%) and 28 (56%), respectively. In hospitalized patients with severe illness and COVID-19, tocilizumab was ineffective in preventing intubation or death. Trials must be larger, however, in order to exclude the potential benefits or harms.

## 1. Introduction

At the end of January 2020, the World Health Organization (WHO) announced the sixth public health emergency outbreak for the coronavirus illness 2019 (COVID-19) [1,2]. The virus has spread fast in China, Japan, and Republic of Korea, with cases confirmed in several countries [3,4]. Fever, cough, myalgia, weariness, dyspnea, and diarrhea are all common symptoms at the start of the disease [5,6]. A percentage of patients end up with pneumonia, which can rapidly lead to respiratory failure and acute respiratory distress syndrome (ARDS) [5,7]. Elderly and immunosuppressed patients are more liable to infection. Therefore, they have a higher mortality rate [8]. According to some research, the death rate for serious cases of ARDS has climbed to 60.5 percent [8]. Interleukin-6 levels are linked to the severity of COVID-19 [9,10]. Interleukin-6 may affect immunological dysregulation and ARDS. In individuals infected with COVID-19, lymphocytes and inflammatory monocytes were found in the pulmonary vasculature, endothelin’s were present, apoptosis occurred, coagulation occurred, and angiogenesis was observed, suggesting that vascular inflammation and dysfunction may play a role in severe pneumonia caused by COVID-19 [11,12].

Interleukin-6 (IL6) may contribute to the vascular dysfunction linked with this illness because it enhances endothelial cell dysfunction and blood vessel permeability [13,14]. Endogenous IL-6 mediates a range of immunological reactions and is produced by inflammatory stimuli. Tocilizumab’s inhibition of IL-6 receptors causes a decrease in the production of cytokines and acute-phase reactants [15]. An acute systemic inflammatory disease called cytokine release syndrome (CRS) is marked by fever and numerous organ failures [16,17]. These characteristics are comparable to cytokine release syndrome (e.g., in response to T-cell immunotherapy). Compared to sepsis and cytokine release syndrome, COVID-19 has much lower amounts of proinflammatory cytokines. Viral evasion of host immunological responses and virus-induced cytopathic consequences. Increased levels of inflammatory cytokines, such as tumor necrosis factor (TNF-α), interleukins (ILs) 2, 7, and 10, granulocyte-colony stimulating factor (G-CSF), monocyte chemoattractant protein 1, macrophage inflammatory protein 1 alpha, and interferon-inducible protein 10, in the plasma of COVID-19 patients, particularly in ICU patients, suggested a cytokine storm had taken place [18], with many interleukin 6, pathogenic T cells, and inflammatory monocytes triggering inflammatory storms. Therefore, a monoclonal antibody that targets the IL-6 pathways may reduce inflammatory storms, leading to clinical outcomes, including a rapid restoration of normal body temperature and enhanced respiratory performance [8]. In order to quiet the inflammatory storm and lower mortality in severe COVID-19 patients, we thus propose that tocilizumab is an effective therapy. Furthermore, tocilizumab is an anti-interleukin-6 receptor alpha monoclonal antibody accustomed to treating inflammatory disorders [19]. As a result, the current study aimed to evaluate tocilizumab’s efficacy in treating severe COVID-19 infection.

## 2. Subjects and Methods

### 2.1. Study Design

**Figure 1:**
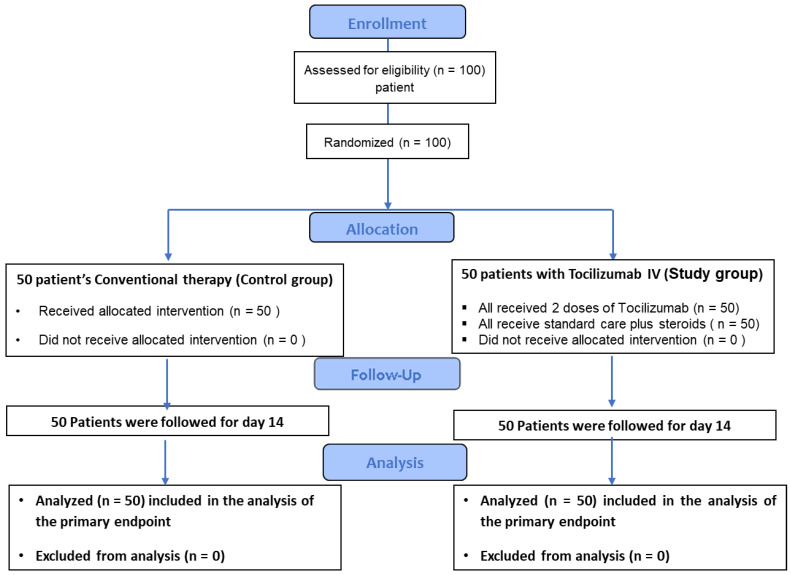
Consort flow diagram.

The open-label randomized controlled clinical trial was conducted from 1 May 2020 to May 2022 to determine the efficacy of tocilizumab (study group B) in the treatment of intensive care for individuals with severe SARS-CoV-2 infection versus conventional therapy (control group A), e.g., any supportive treatment with or without steroids. This study was carried out at Hospital AL-Safwa, Fayum, Egypt. It is one of the reference facilities dedicated to treating severe COVID-19 cases in town. In addition, an outpatient clinic and a 50-bed facility are available. Clinical Trial Number NCT0487185 was assigned to this study when it was launched. The trial was conducted in a manner that was compliant with the standards stated in the Declaration of Helsinki and the standards outlined by the International Conference on Harmonization for Good Clinical Practice. The Research Ethical Committee gave its stamp of approval to the procedure. The trial results were reported following the guidelines provided by the Integrated Guidelines of Reporting Trials (Consort). A web-based approach by Urbaniak and Plous [20] with an assignment ratio of 1:1 was used to randomly choose 100 patients who were eligible for the study. The randomization was divided into strata according to the center (Figure 1). The institutional review board and the ethical committee reviewed the protocol before it was approved.

### 2.2. Participants

Patients were eligible if they met one of the following inclusion criteria: (1) age > 18 years at the time of participation; (2) clinical diagnosis impression of COVID-19, previous record of fever, and any respiratory manifestations, such as cough or shortness of breath, and a SpO_2_ equal to or below 92 percent while breathing indoor air, using supplementation invasive mechanical ventilation (IMV); (3) radiological by computed tomography ground-glass opacity or pulmonary consolidation or chest X-ray demonstrating pulmonary infiltration when the temperature was above 38 °C for more than 72 h (CXR); (4) laboratory-confirmed diagnosis of COVID-19 pneumonia, and a positive rt-PCR assay for SARS-CoV-2 in respiratory tract samples was used after a clinical diagnosis of COVID-19 infection; (5) severe COVID-19 infection diagnosed by several vital sign indicators, including a temperature greater than 38 °C at any point during the last two days, acute respiratory failure with a PaO_2_/FIO_2_ ratio of 200 to 300 mm/Hg (partial pressure of arterial oxygen to fraction of inspired oxygen). When registering, we included patients using venturi masks or high-flow nasal cannulas with preset and recorded FIO_2_ and patients using invasive or noninvasive mechanical ventilation. Invasive or noninvasive mechanical ventilation was also allowed for the patients. An arterial blood gas study was performed on each subject to assess the PaO_2_/FIO_2_ ratio. The inflammation phenotypic features serum levels of C-reactive protein (CRP) over 10 mg/dL, or CRP levels that have risen to at least twice the level at admission were used. The trial comprised patients who required ICU care and had symptoms of moderate to severe COVID-19, at least two of the following: A chest X-ray demonstrating pulmonary infiltration when the temperature was above 38 °C for more than 72 h (CXR). Insufficient oxygen in the blood to maintain a saturation level of more than 92 percent and at least one of the following: CRP > 50 mg/L, ferritin > 500 ng/mL, LDH > 250 U/L, and D-dimer > 1000 ng/mL. The study excluded patients who did not meet the criteria. (1) Children under 18 were excluded from the study due to the documented lower morbidity and mortality rates associated with COVID-19 [21]. (2) In addition, patients with a history of hypersensitivity to tocilizumab, HIV/AIDS, immunosuppressive medicines, pregnant or breastfeeding, decompensated cirrhosis or chronic renal failure, active tuberculosis, or any of the other disorders specified above were not allowed to participate in the trial. (3) All patients and the legal representatives of those unable to consent were provided information on the study’s goals and the risks of participating. They were given ample opportunity to review a document before signing it as their informed consent (ICF). Once they had healed, patients signed their ICF records. (4) Subjects with heart failure defined as NYHA Class III or IV and between the ages of 79 and 86 were excluded from the study. (5) Patients were ineligible for the study if they received intravenous tocilizumab for an ailment other than COVID within three weeks of their first COVID symptom. (6) If the ANC was below 500, the platelets were below 50,000, or the AST/ALT was above five times the upper limit of normal, you were also excluded. The patients were given conventional treatment and a single intravenous infusion of tocilizumab (8 mg per Kg of body weight, with a starting dosage of 400 mg and a maximum dose of 800 mg), followed by a second dose 12–24 h later. According to the treatment guidelines, supportive care was provided to the control arm patients. The drugs allowed as conventional therapy aminoquinolines (hydroxychloroquine), antiviral (remdesivir), antibiotics (azithromycin, ceftriaxone, cefotaxim, and meropnam), glucocorticoids (hydrocortisone, dexamesazone, and methylprednisolone), vitamins (vitamin C and vitamin D), minerals (zinc), and NSAIDs (naproxen and paracetamol). When TCZ was not available or any of the exclusion criteria listed above were present, the local protocol considered the use of corticosteroids for patients with severe COVID-19 pneumonia who met the same inclusion criteria established for the commencement of TCZ. When TCZ was not available or any of the exclusion criteria listed above were present, the local protocol considered the use of corticosteroids for patients with severe COVID-19 pneumonia who met the same inclusion criteria as those for the start of the treatment. The major dosing regimens studied were pulses of 250 mg IV methylprednisolone given over a period of 3 days. Regardless of the later administration of TCZ, the prescription of corticosteroids was generalized for patients coming to the ICU with severe COVID-19. Eight milligrams of dexamethasone were given intravenously or orally once a day for up to 10 days (or until hospital discharge if sooner).

Tumor necrosis factor inhibitors and interleukin-1 inhibitors were not permitted, although steroids were. Except for those two categories, all drugs were authorized. In addition, individuals randomized in either arm of the study were entitled to receive any medication, including tocilizumab, in the case of patients assigned to the control arm if their clinical status worsened.

The term “baseline” refers to the most recent observation made before receiving the tocilizumab or the usual medication on day 1. The patient’s medical status was ranked from one to eight points on an ordinal scale. For the evaluation of patients in this trial, the baseline was defined as the last observation before administering tocilizumab or conventional therapy on day 1. The patients’ clinical status was assessed on eight points ordinal scale according to the following categories: 0, no clinical virology or evidence of infection; 1 and 2, discharged or ready for discharge with no limitation of activities; 3, hospitalization in a nonintensive care unit (ICU) without supplemental oxygen; 4, non-ICU hospitalization with supplemental oxygen; 5, ICU or non-ICU hospitalization with noninvasive ventilation or high-flow oxygen; 6, ICU hospitalization with intubation and mechanical ventilation; 7, ICU hospitalization with extracorporeal membrane oxygenation or mechanical ventilation and additional organ support; and 8, death. Clinical status was recorded at baseline and every day during hospitalization.

### 2.3. Outcomes

The primary objective of this trial was to compare the efficacy of early tocilizumab delivery with conventional therapy. Time-to-event analysis was used to evaluate the main outcome: intubation (or death for patients who died before intubation) after the delivery of tocilizumab or a placebo. The primary endpoint assessed clinical deterioration within seven days of randomization on an ordinal scale of one to eight points. Clinical status was defined as the occurrence of one of the following events: death from any cause, clinical worsening (MV) primary endpoint on an ordinal scale ranging from 1 (discharged or ready for discharge) to 8 (death) in the modified intention-to-treat population, which included all the patients who had received at least one dose of tocilizumab or placebo. Initially, mechanical ventilation was performed when the PaO_2_/FIO_2_ ratios were less than 150 mm Hg, respiratory rates were greater than 30 breaths/min, and signs of respiratory distress or multiorgan failure were present. One of the secondary outcomes was to determine how long a patient would need to be on a mechanical ventilator, the length of stay, and the time to event, death, or mechanical ventilator. Tocilizumab has also been linked to mortality and other serious side effects. The patients who had not been receiving mechanical ventilation at the time of the randomization started receiving mechanical ventilation. The patients who received mechanical ventilation or were treated in the intensive care unit at baseline were defined as dead, starting mechanical ventilation, or worsening clinically for one category. The adverse events were graded according to the Common Terminology Criteria for Adverse Events, version 5.0. Finally, the researchers looked at what could have caused the serious side effects [22].

### 2.4. Statistical Analysis

The modified intention-to-treat group included all patients who had been randomly assigned to receive tocilizumab or conventional medicine, which was used to evaluate the effectiveness of the outcomes. A sample size of 100 patients would have an 80% power to detect a difference in the primary outcome (clinical status at day 14). In a nutshell, the primary analysis was carried out on the ITT population. Furthermore, the patients’ proportion in each arm who experienced a clinical worsening during the two weeks following randomization was compared using the Mann–Whitney test. To compare two or more categorical variables, the Chi-squared test was used. A *p*-value < 0.05 was recognized as significant. The coefficient values (β) and corresponding *p*-values were used to show the results of the linear regressions [23]. Finally, using Kaplan–Meier estimates, we compared the survival curves for mortality and the composite endpoint, and the log-rank test was used to determine statistical significance. SPSS version 28 and SAS version 9.4 (SAS Institute (Carrey, NC, USA)) were used for statistical analysis [24,25].

## 3. Results

This study comprised a total of 100 subjects with COVID-19. The baseline demographic and clinical features at the start of the study (Table 1 and Table 2).

There was no difference in the time of clinical deterioration death or MV (Table 3). Male patients comprised the bulk of the patients (67 percent). One hundred patients were enrolled for the intention-to-treat analysis; fifty were randomized to receive tocilizumab at enrollment and fifty to conventional treatment until clinical deterioration (Figure 1). Fifty patients (100%) in the tocilizumab arm received therapy following the protocol after randomization. According to the study protocol, all patients were tracked for a one-category worsening in clinical status at 14 days and at least 28 days for secondary endpoints. The key composite endpoint between the control and trial groups at 14 days of death was not significantly different in the ITT analysis, with 22.0 percent (n = 11) in the tocilizumab study group A vs. 24.0 percent (n = 12) in the conventional study control group B (Table 4). On the other hand, there was a significant difference in the tocilizumab group with an increase in mechanical ventilators, with 56 percent (n = 28) in study group A vs. 34 percent (n = 17) in control group B (Table 4).

**Figure 2:**
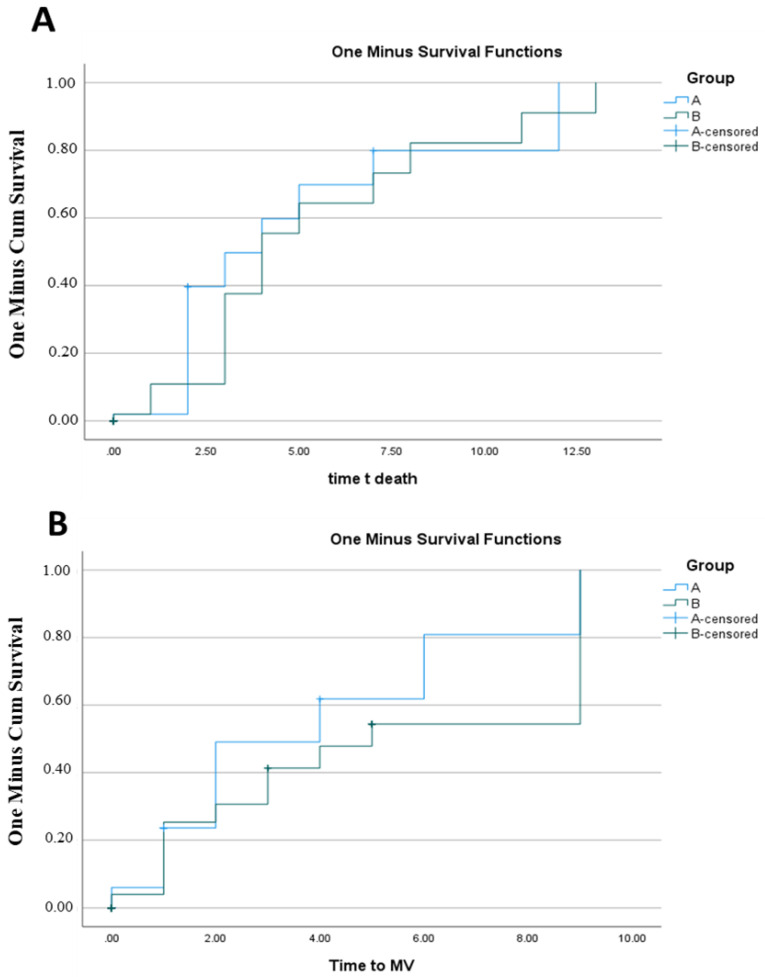
Kaplan–Meier estimates of the cumulative survival time to death (**A**) and mechanical ventilation (**B**).

One patient in the tocilizumab group study developed giant cell arteritis, which was classified as a severe side effect. A total of 17 out of 50 patients (28.0%) in the tocilizumab arm and 17 out of 63 (27.0%) in the conventional treatment arm showed clinical deterioration within 14 days of randomization. Figure 2A shows that all deaths occurred within 5.181 days of randomization, with no differences in time to death between the two arms (*p*-value > 0.05). Figure 2B demonstrates that there were no changes in the time to the occurrence between the two groups and that all 45 incidences of clinical worsening MV occurred within 4.683 days of randomization (*p*-value > 0.05). A total of 43 (43%) of the 100 patients were discharged from the hospital (Table 4). The tocilizumab group B had more discharges (Table 4). The tocilizumab group had a larger number of patients in the hospital for 7 days compared to 4 days in the control group A, with a *p*-value of 0.01 (Table 3). There was no difference between the two groups when it came to the cessation of oxygen requirements. There was a link between CRP and time to death, as well as MV and MV duration (Table 5). The duration of MV was also affected by age (Table 6).

## 4. Discussion

According to observational studies, tocilizumab successfully lowers mortality and/or intubation in patients with severe COVID-19 pneumonia. These encouraging outcomes prompted the conduct of this randomized clinical study. 

Interestingly, the current study’s results reported a significant difference with increased mechanical ventilators in the tocilizumab group. Similarly, tocilizumab did not lower short-term mortality, according to a systematic review and meta-analysis that included five RCTs and 18 cohorts. Cohort studies provide inconclusive evidence that tocilizumab and decreased mortality are related [26]. Another trial revealed that tocilizumab did not reduce mortality in patients on mechanical ventilation who had COVID-19 pneumonia [27].

On the other hand, it has been shown that tocilizumab decreased the likelihood of mechanical ventilation in COVID-19 patients who were hospitalized [26,28,29]. Additionally, a meta-analysis study’s findings showed a significant difference in mortality between the tocilizumab group and the control group in a fixed-effect model (*p* < 0.001), indicating the effectiveness of tocilizumab therapy for severe COVID-19 [30]. Including various patient populations, inflammatory conditions, time of administration, and tocilizumab dosage may be responsible for the disparity in the findings [26]. 

Furthermore, our randomized controlled study revealed that this intervention had no appreciable impact on any of the effective outcomes we looked at, including the risk of intubation or death, the progression of the illness, or the time it took to stop using supplemental oxygen. On the contrary, adding tocilizumab to the standard therapy might decrease mortality in severe COVID-19 [31,32,33].

Regarding the current study, tocilizumab group B had more hospital days (7 days versus 4 days) than control group A with a high significance at a *p*-value < 0.001. In contrast, another observational study reported the absence of any clinical benefit of using tocilizumab on the length of the hospital stay in patients hospitalized with COVID-19 [34]. In addition, another study revealed that severe COVID-19 patients admitted to the ICU did not benefit from tocilizumab in terms of survival and hospital stay due to the delay in the administration of tocilizumab [35]. Thus, the correct timing of the administration of tocilizumab is an important issue for consideration. It improves the effectiveness when administered early at the first signs of clinical worsening [35,36,37]. The conflicting results regarding tocilizumab’s clinical efficacy in severe COVID-19 patients may be related to the difference in randomized and real-life clinical trial enrollment criteria [35].

Our findings support the notion that patients with greater baseline serum CRP concentrations are more likely to have a negative outcome. Even though tocilizumab was ineffective in this study, the population it was tested on, which was composed of several coexisting illnesses, did not experience an overwhelming amount of high-grade adverse effects. However, given the confidence intervals for our efficacy comparisons, we cannot rule out the potential that tocilizumab therapy may have some positive or negative effects on certain patients. Therefore, our findings do not support the idea that early interleukin-6 receptor blockage is a successful therapeutic approach in critically sick COVID-19 hospitalized patients. The premise of our study was that interleukin-6 receptor blocking, when administered to patients with sickness that had not yet resulted in intubation, would stop the cytokine storm brought on by COVID-19 and avert the worst effects of the disease.

At the same time, another study hypothesized that COVID-19 induced cytokine storm together with the concomitant nonreversible multiorgan injury. However, at this stage, a full-blown cytokine storm is dependent on multiple immunological pathways, and the inhibition of IL-6 appears insufficient to cure the disease [35]. 

CRP is the sole diagnostic marker shared by all published studies, and cytokine storm is defined as having levels of 5–10 mg/dL (10–20 times the upper limits of normal) [36]. This research revealed a significant difference in CRP levels between the tocilizumab and the control groups. In agreement with our results, it has been documented that CRP was reduced in all patients following tocilizumab administration, as seen in previous reports of tocilizumab and an IL-1 receptor antagonist [36,38,39,40]. According to cumulative evidence of intermediate confidence, tocilizumab lowers the likelihood that COVID-19 patients in hospitals will need mechanical breathing [41]. Despite tocilizumab’s failure to cut short-term mortality in RCTs, low-certainty data from cohort studies point to a possible link between the drug and reduced mortality [42]. Although the findings of observational studies and the impact of tocilizumab on mortality are incongruent, there are several other possibilities outside study design and residual confounding that might be the cause. These factors include the various patient demographics, the degree of inflammation, and the time of tocilizumab administration and dosage. Additionally, since the RCTs did not include death as a separate main endpoint, they lacked the capacity to distinguish between the effects of tocilizumab and the control on mortality [43].

Finally, the difference in the results of the performance of tocilizumab in severe COVID-19 patients may be attributed to different factors, such as its co-administration with corticosteroids and its immunomodulatory effect. Moreover, the selection of the right patients and the correct timing of the administration of tocilizumab influence its efficacy. 

### Limitations

Our trial offered certain advantages as well as some limitations. The open-label nature of the experiment is one of its drawbacks. There was a possibility concerning whether a double-blind, placebo-controlled experiment could be conducted. However, it was disregarded for two primary reasons: tocilizumab’s inherent anti-IL-6 action and the logistical challenges of coordinating it during a crisis. In patients with COVID-19, it has been repeatedly documented and clinically seen that this monoclonal antibody quickly reduces fever and blood CRP levels.

## 5. Conclusions

Our study observed the absence of clear improvements in patients receiving tocilizumab compared to standard management. Therefore, in our opinion, the efficacy of tocilizumab in severe COVID-19 depends on the selection of the right patients and the right administration time. Thus, further studies should be conducted focusing on the timing of the administration of tocilizumab and its co-administration with corticosteroids in severe COVID-19 patients.

## Figures and Tables

**Table 1:** Demographic and Clinical Characteristics of the Patients at Baseline.

	Group A (Conventional) N = 50	Group B (Tocilizumab) N = 50	Mann–Whitney U Statistic	*p*-Value
Age, median (IQR), years	63.00 (55.75–66.0)	66.00 (55.0–70.0)	1140.50	0.44 NS
Weight, kg	80.0 (67.00–80.00)	70.00 (60.00–88.50)	1020.50	0.111 NS
Height, (meter)	1.68 (1.60–1.70)	1.66 (1.62–1.70)	1237.50	0.932 NS
BMI, kg/m^2^	27.68 (26.72–28.89)	26.64 (23.43–30.65)	996.50	0.080 NS
RR median (IQR), breaths/min	26.00 (24.00–30.00)	22.00 (21.00–30.65)	493.00	<0.001 HS
FIO_2_, mm Hg	0.21 (0.21–0.21)	0.21 (0.21–0.21)	1125.00	0.166 NS
PAO_2_, mm Hg	56.0 (55.00–73.00)	66.00 (49.25–73.00)	1239.50	0.94 NS
Max. temp, median (IQR), °C	38.00 (38.00–38.00)	37.90 (37.50–38.00)	845	0.002 s
Systolic BP, mm Hg	120.00 (110.00–132.50)	130.0 (127.50–140.0)	841	0.004 S
Diastolic, mm Hg	80.00 (70.00–90.00)	85.00 (80.00–100.00)	837.000	0.003 S
HR	88.00 (85.00–92.00)	99.00 (90.0–103.00)	462.50	<0.001 HS
GCS at admission	15.00 (15.00–15.00)	15.00 (15.00–15.00)	1210	0.61 NS
IL-6, median (IQR), pg/mL	36.18 (17.25–58.25)	51.05 (29.25–65.52)	977.00	0.060 NS
Comorbidities Diabetes CKD IHD COPD Hypertension	20(40.0%) 3(6.0%) 6(12.0%) 0(0.0%) 26(52.0%)	22(44.0%) 0(0.0%) 16(32.0%) 5(10.0%) 22(44.0%)	X^2^ = 0.164 X^2^ = 3.093 X^2^ = 5.828 X^2^ = 5.263 X^2^ = 0.641	0.685 NS 0.079 NS 0.016 S 0.022 S 0.423 NS

NS: nonsignificant at a *p*-value > 0.05; S: significant at a *p*-value < 0.05; HS: highly significant at a *p*-value < 0.001.

**Table 2:** Laboratory Characteristics of the Studied Patients at Baseline.

	Group A (Conventional) N = 50	Group B (Tocilizumab) N = 50	Mann–Whitney U Statistic	*p*-Value
CRP, mg/dL	103.0 (100.00–120.0)	104.00 (72.0–108.25)	913.00	0.019 S
Creatinine	1.50 (1.40–1.90)	1.40 (1.20–1.90)	1020	0.111 NS
HB	13.00 (11.70–15.17)	12.00 (11.40–13.72)	999.50	0.084 NS
TLC/μL	8400.0 (6000–18,750)	11450.0 (10,175–14,825)	1052.50	0.174 NS
Lymphocyte Count, %	12.00 (10.00–15.00)	10.00 (6.00–15.00)	985.00	0.064 NS
Neutrophile Count, %	80.00 (80.00–82.50)	84.00 (80.00–87.75)	888.00	0.011 S
NLR, %	6.83 (5.33–8.30)	8.30 (5.39–14.49)	963	0.047 S
PLT ×10^3^/μL	281.0 (187.0–332.5)	246.5 (186.0–291.50)	1074.50	0.227 NS
NA	141.50 (138.00–143.0)	140.000 (137.0–143.0)	1122.50	0.379 NS
K	4.00 (3.80–4.30)	4.20 (3.94–4.43)	1049.50	0.167 Ns
Temp, °C	38.00 (38.00–38.0)	38.00 (38.00–38.0)	1250.00	1.00 NS
Ferritin, ng/mL	564.00 (500.0–758.0)	721.0 (486.25–1500)	997.00	0.080 NS
AST	33.50 (25.00–41.75)	55.00 (45.00–71.00)	4325.50	<0.001 HS
LDH	576.50 (441.00–825.75)	881.00 (718.00–1147.50)	506.00	<0.001 HS
D-Dimer, ng/mL	463.50 (283.00–1001.0)	513.0 (251.0–1014.0)	1236.50	0.928 NS
ALT	37.50 (25.00–40.00)	55.00 (40.00–88.00)	568.500	<0.001 HS
O_2_ saturation at admission	80.00 (70.00–85.00)	80.00 (70.00–89.00)	1135.50	0.429 NS

NS: nonsignificant at a *p*-value > 0.05; S: significant at a *p*-value < 0.05; HS: highly significant at a *p*-value < 0.001.

**Table 3:** Clinical Outcomes in the Intention-to-Treat Population Time analysis.

	Group A (Conventional) N = 50	Group B (Tocilizumab) N = 50	Mann–Whitney U Statistic	*p*-Value
Time to death	0.00 (0.00–2.00)	0.00 (0.00–0.00)	1225.0	0.825 NS
Time to MV	0.00 (0.00–1.00)	0.00 (0.00–3.00)	1138.50	0.364 NS
Duration of MV days	0.00 (0.00–2.00)	1.50 (0.00–3.00)	1027.00	0.098 NS
Patient hospital days	4.00 (3.00–6.00)	7.00 (4.75–10.00)	564.50	<0.001 HS

NS: nonsignificant at a *p*-value > 0.05; HS: highly significant at a *p*-value < 0.001.

**Table 4:** Overall events outcome.

Outcomes	Group A (Conventional) N = 50	Group B (Tocilizumab) N = 50	Chi-Square	*p*-Value
Clinical worsening (MV) primary endpoint	17 (34%)	28 (56%)	4.889	0.027 S
Deaths at the end of follow-up	11 (22%)	12 (24%)	0.056	0.812 NS
Discharges at the end of follow-up	19 (38%)	24 (48%)	25.5	0.001 S

NS: nonsignificant at a *p*-value > 0.05; S: significant at a *p*-value < 0.05.

**Table 5:** The result of the linear regression between CRP and confirmed COVID-19 cases.

CRP	Unstandardized Coefficients		Standardized Coefficient	t	Sig.
	B	Std. Error	Beta		
(Constant)	2256.774	813.942		2.773	0.008
Time at death	−8.036	2.980	−0.988	−2.696	0.010
Time to MV	6.762	3.162	0.620	2.139	0.038
Duration of MV days	8.850	3.948	0.833	2.242	0.030

MV: mechanical ventilation.

**Table 6:** The result of the linear regression between age and confirmed COVID-19 cases.

Age	Unstandardized Coefficients		Standardized Coefficient	t	Sig.
	B	Std. Error	Beta		
(Constant)	688.895	297.89		2.313	0.026
Duration of MV days	−5.172-	1.406	−1.094-	−3.677-	<0.001

MV, mechanical ventilation.

## Data Availability

Data sharing is not applicable to this article, as no data sets were generated or analyzed during the current study. All data given are available in the original articles.
